# Air pollution is associated with faster cognitive decline in Alzheimer's disease

**DOI:** 10.1002/acn3.51779

**Published:** 2023-04-27

**Authors:** Young‐gun Lee, Seon‐Jin Yoon, So Hoon Yoon, Sung Woo Kang, Seun Jeon, Minseok Kim, Dong Ah Shin, Chung Mo Nam, Byoung Seok Ye

**Affiliations:** ^1^ Department of Neurology Yonsei University College of Medicine Seoul South Korea; ^2^ Department of Neurology, Ilsan Paik Hospital Inje University College of Medicine Goyang South Korea; ^3^ Department of Neurosurgery Yonsei University College of Medicine Seoul South Korea; ^4^ Department of Biostatistics and Computing Yonsei University College of Medicine Seoul South Korea; ^5^ Department of Preventive Medicine Yonsei University College of Medicine Seoul South Korea

## Abstract

**Objective:**

Although chronic exposure to air pollution is associated with an increased risk of dementia in normal elderlies, the effect of chronic exposure to air pollution on the rates of cognitive decline in Alzheimer's disease (AD) has not been elucidated.

**Methods:**

In this longitudinal study, a total of 269 patients with mild cognitive impairment or early dementia due to AD with the evidence of brain β‐amyloid deposition were followed‐up for a mean period of 4 years. Five‐year normalized hourly cumulative exposure value of each air pollutant, such as carbon monoxide (CO), nitrogen dioxide (NO_2_), sulfur dioxide (SO_2_), and particulate matter (PM_2.5_ and PM_10_), was computed based on nationwide air pollution database. The effects of chronic exposure to air pollution on longitudinal cognitive decline rate were evaluated using linear mixed models.

**Results:**

Higher chronic exposure to SO_2_ was associated with a faster decline in memory score, whereas chronic exposure to CO, NO_2_, and PM_10_ were not associated with the rate of cognitive decline. Higher chronic exposure to PM_2.5_ was associated with a faster decline in visuospatial score in apolipoprotein E ε4 carriers. These effects remained significant even after adjusting for potential confounders.

**Interpretation:**

Our findings suggest that chronic exposure to SO_2_ and PM_2.5_ is associated with faster clinical progression in AD.

## Introduction

Several epidemiological studies have revealed an association between chronic exposure to air pollution and the risk of developing dementia.^1^, ^2^, ^3^, ^4^, ^5^, ^6^, ^7^, ^8^, ^9^ In specific, chronic exposure to fine (<2.5 μm) and coarse (<10 μm) particulate matter (PM_2.5_ and PM_10_) and nitrogen dioxide (NO_2_) is associated with faster cognitive decline in normal elderly population.^10^, ^11^, ^12^, ^13^ Multiple air pollutants, including PM, NO_2_, sulfur dioxide (SO_2_), and carbon monoxide (CO), are neurotoxic,^14^ by increasing oxidative stress, disrupting integrity of blood–brain–barrier (BBB). Based on this evidence, the Lancet Commission added air pollution as a potential modifiable risk factor for dementia in 2020,^15^ and emphasize that the management of air pollution could reduce the risk of developing dementia.

The longitudinal effects of air pollution on cognitive decline in patients with AD remain unclear. One study reported that neuropathologic burden of AD are increased in those with chronic exposure to air pollution.^16^ A study based on two cities in Taiwan showed that CO, NO_2_, SO_2_, and PM_10_ exposure was associated with faster clinical deterioration in AD.^17^ However, another study based in the United States showed no effect of PM_2.5_ on longitudinal decline in cognitively impaired patients.^18^ As previous studies lacked detailed neuropsychological evaluation and did not provide evidence related to biomarkers of Aβ deposition, it is not clear specifically which air pollutants are associated with cognitive decline in AD. Furthermore, comprehensive analysis of multiple air pollutants was not conducted.

In the present study, we aimed to investigate the impact of air pollutants on cognitive deterioration based on longitudinal neuropsychological test in 269 patients with clinical AD with brain Aβ deposition. We hypothesized that chronic exposure to air pollutants would be associated with faster cognitive decline in patients with AD. By using 5‐year normalized hourly cumulative exposure to air pollutants for each participant, we focused on the chronic effects of air pollutants to suggest a target for both clinical and policy‐making perspectives.

## Methods

### Study participants

**Figure 1:**
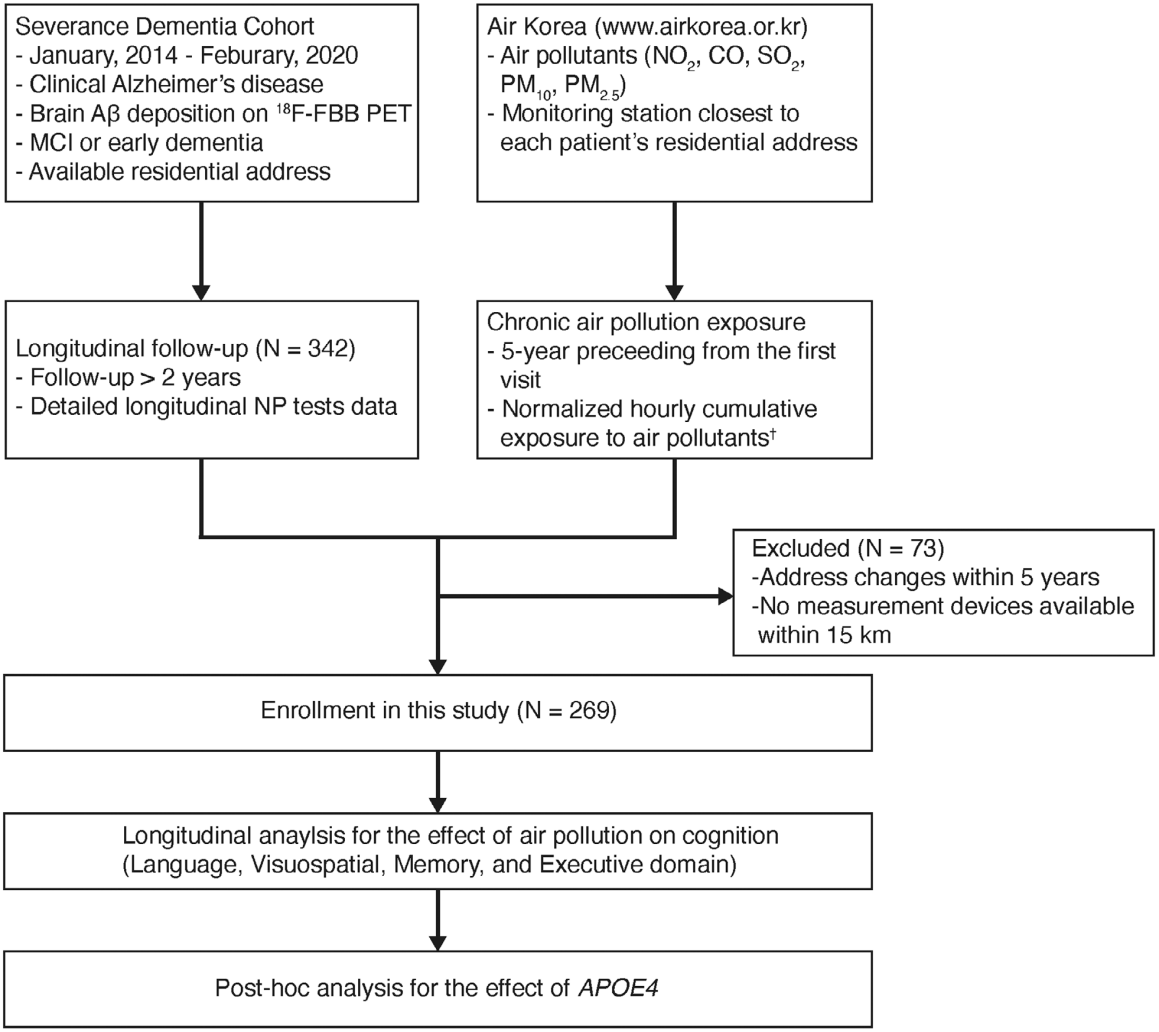
Participant selection flowchart. Aβ, β‐amyloid; *APOE4*, apolipoprotein E ε4; CO, carbon monoxide; ^18^F‐FBB, ^18^F‐florbetaben; NO_2_, nitrogen dioxide; MCI, mild cognitive impairment; NP, neuropsychological; PET, positron emission tomography, PM, particulate matter; SO_2_, sulfur dioxide. ^†^Normalized hourly cumulative value = $\sum_{t_{i}=1}^{24\times 365\times 5\;\text{hours}}\frac{\text{Pollutant}\left(i,t_{i}\right)\times \Delta t}{\sum \Delta t.}$.

This study included 342 consecutive patients of AD who first visited the Dementia Outpatient Clinic at Severance Hospital, Yonsei University Health System, between January, 2014 and February, 2020. The patients were clinically diagnosed as AD with brain Aβ deposition confirmed by ^18^F‐florbetaben (FBB) positron emission tomography (PET). Clinical stage of the patients were mild cognitive impairment (MCI) or early dementia with Clinical Dementia Rating^®^ (CDR) either 0.5 or 1, respectively. Subjects were followed‐up for more than 2 years and underwent detailed neuropsychological testing at least twice. Seventy‐three patients were excluded, as their addresses had changed within the 5 years preceding baseline or the air pollutant monitoring station was farther than 15 km from their address. Therefore, 269 patients were included in the final analysis (Fig. 1). Information regarding previous occupational history, smoking and alcohol history, body mass index (BMI), comorbidities—including hypertension, type 2 diabetes, dyslipidemia, and stroke—and apolipoprotein E ε4 (*APOE4*) carrier status was obtained based on a review of patient medical records.

### Standard protocol approval, registration, and patient consent

This study was approved by the Institutional Review Board of Severance Hospital (IRB 4‐2022‐0221); the requirement for informed consent was waived because this study was based on a retrospective chart review.

### Clinical assessment and neuropsychological evaluation

All participants underwent testing using the standardized Seoul Neuropsychological Screening Battery 2nd Edition (SNSB‐II),^19^, ^20^ at their first visit and during the follow‐up period. This battery includes the following scorable tests: the digit span test (forward and backward), Korean version of the Boston Naming Test (K‐BNT), Seoul Verbal Learning Test (SVLT; includes immediate recall, 20‐min delayed recall, and recognition tests), Rey‐Osterrieth Complex Figure Test (RCFT; copying, immediate recall, 20‐min delayed recall, and recognition tests), Clock‐Drawing Test (CDT), set‐shifting ability (go‐no‐go), phonemic and semantic Controlled Oral Word Association Test (COWAT), Stroop Word Reading (Stroop‐WR), Stroop Color Reading (Stroop‐CR), Digit Symbol Coding (DSC), and Korean‐Trail Making Test (K‐TMT).^21^ Age‐ and education‐specific norms were based on 1,067 overall and cognitively healthy community‐dwelling individuals. General cognitive status was assessed using the Korean version of the Mini‐Mental Status Examination (K‐MMSE) and the CDR.

Normally distributed test scores, such as the K‐BNT, SVLT, RCFT, COWAT, Stroop‐CR, DSC, and K‐TMT scores, were z‐transformed using the mean and standard deviation values of the 1,067 norms. Scores that did not follow a normal distribution, such as the CDT and go‐no‐go results, were standardized using percentile scores. The composite scores of the language, visuospatial, memory, and executive domains were calculated using the average of the standardized scores of the corresponding test. Specifically, the language domain composite score was based on the K‐BNT; the visuospatial domain score on the copying items of the RCFT and CDT; the memory domain score on the immediate recall, delayed recall, and recognition tests of the SVLT and RCFT; and the executive domain score on the go‐no‐go, phonemic COWAT, Stroop‐CR, DSC, and K‐TMT part B.

### Patient exposure to air pollutants

We calculated patient exposure to air pollutants using publicly available Air Korea (www.airkorea.or.kr, Korea Environment Corporation) data. Monitoring station‐wise data sets were converted to patient‐wise data sets using a previously reported algorithm.^22^ Briefly, we extracted the monitoring station closest to each patient's residential address from the Air Korea dataset. PM_2.5_ measurements were not available for 18 patients, who were therefore excluded from all statistical analyses for PM_2.5_. We calculated prehospital visit pollutant exposure of 5 years preceding from the date of the first visit to the outpatient clinic of our hospital between January, 2014 and February, 2020. We included exposure data if the measurement value was more than zero (negative or zero values were excluded during the preprocessing step, and these exposure data were not included during the averaging algorithm). All 5‐year cumulative values of hourly exposure were divided by the hour duration of valid pollutant measurements to obtain the normalized average pollutant exposure for a patient (Fig. S1). We excluded cases in which the patient reported house movement during the continuous exposure time‐window of the prehospital visit.

### Global Aβ burden measurement using ^**18**^F‐florbetaben PET imaging

FBB PET was performed using a Discovery 600 platform (General Electric Healthcare, Milwaukee, MI, USA). The FBB PET images were acquired 90 min after the administration of 300 MBq (8 mCi) FBB for 20 min. Images were acquired with a 256 × 256 matrix and reconstructed with an ordered‐subsets expectation–maximization algorithm in iso‐0.98‐mm voxel size. We measured the global FBB PET standardized uptake value ratio (SUVR) using a MATLAB‐based MRI‐free pipeline as previously described.^23^ Briefly, individual scans were registered nonlinearly to an FBB PET template generated by the Alzheimer's Disease Neuroimaging Initiative. We then estimated the full width at half maximum (FWHM) of an individual scan to apply a differential smoothing kernel with a target resolution of 10 mm^3^ using the AFNI 3dFWHMx function.^24^ Finally, using the standard Global Alzheimer's Association Interactive Network (http://www.gaain.org/centiloid‐project) volumes‐of‐interest (VOIs), we quantified individual global SUVRs using cortical VOIs as a target and cerebellar gray matter VOIs as a reference region. We (Y. Lee, S. Jeon, and B.S. Ye) visually inspected the automated pipeline outcomes for quality control and corrected minor registration defects manually.

### Statistical analyses

Statistical analyses of the demographic and clinical data were performed using R version 4.1.0 (R Foundation for Statistical Computing, Vienna, Austria). General linear models were used to evaluate the effects of chronic exposure to air pollutants on baseline cognitive scores. Five‐year normalized hourly cumulative value for each air pollutant was used as a predictor. Age, sex, education, *APOE4* carrier status, and baseline CDR were included as covariates in Model 1 and occupational history, insurance type, smoking and alcohol history, and BMI were further included as covariates in Model 2. Thereafter, comorbidities, including hypertension, type 2 diabetes, dyslipidemia, and stroke, were further added as covariates in Model 3.

To evaluate the effect of chronic exposure to air pollutants on the rate of cognitive declines, we used linear mixed models (LMMs) for domain‐specific cognitive scores using 5‐year normalized hourly cumulative value for each air pollutant (exposure), follow‐up years from baseline (time), and interaction between the exposure and time terms (exposure × time) were used as predictors. Random intercepts and random slopes were included in the models to allow the subject specific cognitive changes. Each exposure × time interaction term was used to measure the effect of 5‐year normalized cumulative exposure to air pollutants on the rate of change of cognitive scores. We first analyzed the effect of each pollutant separately in a single‐pollutant model. The same covariates of general linear models were used in Models 1–3. Then multiple pollutants were simultaneously included in a multi‐pollutant model with the same covariates. To avoid multicollinearity, if a pair of cumulative exposure of air pollutants were highly correlated (r > 0.7) (Table S1), one of the variables were excluded in the multi‐pollutant models. In the sensitivity analysis, we treated 5‐year normalized hourly cumulative values of chronic exposure to air pollutants as categorical variables based on quartile distribution. In each analysis, the false discovery rate method was used to correct for multiple comparisons across the five air pollutants. To evaluate the independent effect of quantified global amyloid deposition on the longitudinal cognitive decalin, we further conducted sensitivity analysis after further consideration of global SUVR in LMMs for domain‐specific cognitive scores.

To investigate the interacting effects of *APOE4* on the relationship between chronic e xposure to air pollutants and longitudinal cognitive decline rate, we further conducted LMMs including three‐way interaction term of *APOE4* × exposure × time as predictors. Then, LMMs for domain‐specific cognitive scores were conducted in the subgroups stratified by *APOE4* carrier status.

## Results

### Participants characteristics

The demographic and clinical characteristics of the study subjects (*n* = 269) are summarized in Table 1. At baseline, the mean age of participants was 74.2 years (SD, 7.0 years), and 63% were female. The mean K‐MMSE total score was 23.5 (SD, 3.2). CDR scores were 0.5 (84%) or 1.0 (16%). During a mean 4.0 years of follow‐up, the participants underwent a median of 2 (range, 2–5) detailed neuropsychological testing with a mean interval of 2.7 years (SD, 1.2 years) between the tests. Five‐year normalized hourly cumulative exposures were as follows: CO, 0.57 parts per million (ppm) (SD, 0.11 ppm); NO_2_, 0.03 ppm (SD, 0.01 ppm); SO_2_, 0.01 ppm (SD, 0.001 ppm); PM_10_, 0.05 ng/m^3^ (SD, 0.01 ng/m^3^) and PM_2.5_, 0.03 ng/m^3^ (SD, 0.003 ng/m^3^). The geographic distributions of each participant and 5‐year normalized hourly cumulative exposure to each air pollutant are depicted in Figure S2.

**Table 1:** Baseline characteristics of the study subjects.

Characteristic	*N* = 269
Age, years	74.2 (±7.0)
Female, no. (%)	170 (63.2%)
Education, years	10.2 (±4.8)
Medical aid type insurance, no. (%)	4 (1.5%)
Follow‐up duration, years	4.0 (±1.3)
Number of cognitive tests, no (range)	2 (2–5)^2^
Interval between cognitive tests, years	2.7 (±1.2)
*APOE4* carriers, no. (%)	144 (53.5%)
K‐MMSE total score	23.5 (±3.2)
CDR, no. (%)	
0.5	227 (84.4%)
1.0	42 (15.6%)
Hypertension, no. (%)	152 (56.5%)
Type 2 diabetes, no. (%)	55 (20.4%)
Dyslipidemia, no. (%)	68 (25.3%)
Stroke, no. (%)	19 (7.1%)
BMI (kg/m^2^)	23.6 (±3.7)
CO (ppm)^1^	0.57 (±0.11)
NO_2_ (ppm)^1^	0.03 (±0.01)
SO_2_ (ppm)^1^	0.01 (±0.001)
PM_10_ (ng/m^3^)^1^	0.05 (±0.01)
PM_2.5_ (ng/m^3^)^1^ ^,^ ^3^	0.03 (±0.003)

Plus‐minus values are mean ± SD.

*APOE4*, apolipoprotein E ε4; BMI, body mass index; CDR, Clinical Dementia Rating®; CO, carbon monoxide; K‐MMSE, Korean version of the Mini‐Mental Status Exam; NO_2_, nitrogen dioxide; PM, particulate matter; SO_2_, sulfur dioxide.

^1^ Normalized hourly cumulative value.

^2^ Median with range.

^3^ *N* = 251.

### Effects of chronic exposure to air pollution on baseline cognitive scores

The 5‐year normalized hourly cumulative exposures to air pollutants were not associated with baseline cognitive scores after adjusting for possible cofounders (Table S2).

### Effects of chronic exposure to air pollution on cognitive decline rates

In single‐pollutant LMMs for longitudinal cognitive scores (Table 2 and Tables S3–S7), we examined the relation of 5‐year normalized hourly cumulative exposure to each air pollutant with the rate of cognitive decline. Higher exposure to SO_2_ was associated with a faster rate of decline in memory domain (*β* = −66.18, 95% confidence interval [CI] = −113.18 to −19.54, *p* = 0.006) after adjusting for age, sex, education, *APOE4* carrier status, and baseline CDR (Model 1). The interacting effect of SO_2_ and time remained significant after adjustment for demographic cofounders (Model 2) and comorbidities (Model 3). Chronic exposure to other air pollutants (CO, NO_2_, PM_10_, and PM_2.5_) were not associated with cognitive decline rates. When amyloid deposition was further considered as covariates, the effect of SO_2_ remained significant (Table S8).

**Table 2:** Effects of chronic exposure to air pollutant on the rate of cognitive declines.

Domain	Predictor	Model 1		Model 2		Model 3	
		*β* (95% CI)	*p*	*β* (95% CI)	*p*	*β* (95% CI)	*p*
Language	CO × year	−0.28 (−0.67–0.10)	0.151	−0.28 (−0.67–0.10)	0.149	−0.29 (−0.67–0.10)	0.145
	NO_2_ × year	−3.58 (−8.53–1.37)	0.157	−3.65 (−8.57–1.30)	0.148	−3.65 (−8.57–1.30)	0.147
	SO_2_ × year	−10.18 (−62.92–42.42)	0.705	−8.56 (−61.13–43.97)	0.750	−8.33 (−60.84–44.22)	0.756
	PM_10_ × year	−5.54 (−13.83–2.74)	0.191	−5.55 (−13.82–2.72)	0.189	−5.54 (−13.80–2.73)	0.190
	PM_2.5_ × year	−12.63 (−28.48–3.39)	0.119	−12.67 (−28.48–3.30)	0.117	−12.73 (−28.54–3.23)	0.115
Visuospatial	CO × year	−0.23 (−1.02–0.56)	0.575	−0.21 (−1.01–0.57)	0.597	−0.21 (−1.00–0.57)	0.596
	NO_2_ × year	5.28 (−4.78–15.43)	0.306	5.15 (−4.84–15.33)	0.316	5.15 (−4.80–15.34)	0.315
	SO_2_ × year	14.53 (−91.92–122.50)	0.789	20.00 (−85.76–128.53)	0.711	19.69 (−85.70–127.97)	0.715
	PM_10_ × year	−8.18 (−25.07–8.62)	0.342	−8.18 (−25.10–8.49)	0.339	−8.25 (−25.17–8.39)	0.335
	PM_2.5_ × year	−32.42 (−64.61–0.67)	0.048	−32.06 (−64.40–0.79)	0.048	−32.24 (−64.72–1.06)	0.047
Memory	CO × year	−0.28 (−0.64–0.07)	0.116	−0.29 (−0.64–0.07)	0.114	−0.28 (−0.64–0.07)	0.116
	NO_2_ × year	−4.08 (−8.58–0.43)	0.078	−4.05 (−8.56–0.47)	0.080	−4.04 (−8.54–0.48)	0.081
	SO_2_ × year	−66.18 (−113.18–19.54)	0.006^1^	−66.22 (−113.36–19.67)	0.006^1^	−66.48 (−113.64–20.05)	0.006^1^
	PM_10_ × year	−3.96 (−11.50–3.62)	0.305	−3.94 (−11.49–3.64)	0.307	−3.97 (−11.52–3.61)	0.304
	PM_2.5_ × year	6.65 (−7.41–20.84)	0.356	6.52 (−7.53–20.72)	0.365	6.46 (−7.59–20.66)	0.369
Executive	CO × year	0.01 (−0.53–0.55)	0.974	0.01 (−0.53–0.56)	0.969	0.01 (−0.53–0.56)	0.971
	NO_2_ × year	1.40 (−5.55–8.34)	0.693	1.38 (−5.57–8.32)	0.698	1.38 (−5.57–8.32)	0.697
	SO_2_ × year	−25.94 (−97.26–45.63)	0.477	−24.47 (−95.91–47.40)	0.503	−24.40 (−95.79–47.46)	0.504
	PM_10_ × year	0.19 (−11.39–11.81)	0.975	0.21 (−11.36–11.85)	0.971	0.22 (−11.35–11.85)	0.971
	PM_2.5_ × year	−2.92 (−24.71–19.15)	0.790	−3.15 (−25.05–18.93)	0.773	−3.16 (−25.07–18.91)	0.773

Data represent the results of linear mixed model analysis for longitudinal cognitive scores using air pollutants, year, and the interaction term between air pollutants and year (air pollutants × year) as predictors. Model 1 was adjusted for age, sex, education, *APOE4* carrier status, and baseline CDR. Model 2: model 1 + further adjusted for the occupational history, insurance type, smoking and alcohol history, and BMI. Model 3: model 2 + further adjusted for comorbidities including hypertension, dyslipidemia, type 2 diabetes, and stroke.

*APOE4*, apolipoprotein E ε4; BMI, body mass index; CDR, Clinical Dementia Rating®; CO, carbon monoxide; NO_2_, nitrogen dioxide; PM, particulate matter; SO_2_, sulfur dioxide.

^1^ Significant after multiple comparison corrections across five air pollutants using the false discovery rate method.

Next, we examined the relation of average 5‐year normalized hourly cumulative exposure to multiple air pollutants simultaneously (Table S9). Higher exposure to SO_2_ was associated with a faster rate of decline in memory domain, while other pollutants were not associated with the cognitive decline rate (Table S9).

In sensitivity analyses, we used quartiles of 5‐year normalized hourly cumulative exposure to each air pollutant (Table 3). Compared to the participants with the lowest SO_2_ exposure quartile group (2.40–4.80 ppb), participants with the highest SO_2_ exposure quartile group (5.70–9.43 ppb) had more rapid decline in memory domain score (*β* = −0.16, 95% CI = −0.27 to −0.04, *p* = 0.008; Fig. S3).

**Table 3:** Effects quartiles of chronic exposure to air pollutants on the rate of cognitive declines.

Predictor	Category	Language domain		Visuospatial domain		Memory domain		Executive domain	
		*β* (95% CI)	*p*	*β* (95% CI)	*p*	*β* (95% CI)	*p*	*β* (95% CI)	*p*
CO	1st quartile [267.54–493.84 ppb]	Reference		Reference		Reference		Reference	
	2nd quartile [493.91–536.96 ppb]	−0.10 (−0.22–0.03)	0.122	0.04 (−0.21–0.30)	0.737	−0.01 (−0.12–0.11)	0.882	−0.06 (−0.24–0.12)	0.499
	3rd quartile [537.26–626.75 ppb]	−0.09 (−0.22–0.03)	0.133	−0.20 (−0.45–0.05)	0.112	0.00 (−0.12–0.11)	0.955	−0.06 (−0.23–0.12)	0.512
	4th quartile [628.0–932.24 ppb]	−0.15 (−0.27–0.02)	0.020	−0.11 (−0.36–0.14)	0.387	−0.08 (−0.20–0.03)	0.145	−0.09 (−0.26–0.08)	0.302
	Interquartile increase^1^	−0.04 (−0.08–0.00)	0.029	−0.06 (−0.14–0.02)	0.159	−0.03 (−0.06–0.01)	0.167	−0.03 (−0.08–0.03)	0.325
NO_2_	1st quartile [9.06–26.17 ppb]	Reference		Reference		Reference		Reference	
	2nd quartile [26.60–31.36 ppb]	−0.05 (−0.17–0.08)	0.471	0.05 (−0.21–0.30)	0.716	−0.04 (−0.16–0.07)	0.472	−0.10 (−0.27–0.08)	0.280
	3rd quartile [31.44–34.57 ppb]	−0.02 (−0.14–0.10)	0.762	−0.03 (−0.27–0.22)	0.819	−0.09 (−0.20–0.02)	0.113	−0.12 (−0.29–0.05)	0.170
	4th quartile [34.58–58.94 ppb]	−0.12 (−0.24–0.01)	0.074	0.13 (−0.12–0.39)	0.299	−0.09 (−0.20–0.03)	0.133	0.00 (−0.17–0.18)	0.977
	Interquartile increase^1^	−0.03 (−0.07–0.01)	0.118	0.03 (−0.05–0.11)	0.442	−0.03 (−0.07–0.00)	0.087	0.00 (−0.06–0.05)	0.944
SO_2_	1st quartile [2.40–4.80 ppb]	Reference		Reference		Reference		Reference	
	2nd quartile [4.81–5.17 ppb]	0.00 (−0.13–0.13)	0.974	0.10 (−0.15–0.36)	0.454	−0.08 (−0.20–0.03)	0.165	−0.04 (−0.22–0.14)	0.649
	3rd quartile [5.18–5.70 ppb]	−0.02 (−0.14–0.11)	0.802	0.00 (−0.26–0.26)	0.985	−0.08 (−0.20–0.03)	0.153	−0.05 (−0.23–0.13)	0.607
	4th quartile [5.70–9.43 ppb]	−0.01 (−0.14–0.12)	0.886	0.15 (−0.10–0.41)	0.255	−0.16 (−0.27–0.04)	0.008^2^	−0.01 (−0.19–0.16)	0.870
	Interquartile increase^1^	0.00 (−0.04–0.04)	0.846	0.04 (−0.04–0.12)	0.380	−0.05 (−0.08–0.01)	0.012	0.00 (−0.06–0.05)	0.892
PM_10_	1st quartile [37.42–44.49 μg/m^3^]	Reference		Reference		Reference		Reference	
	2nd quartile [44.50–47.43 μg/m^3^]	0.11 (−0.02–0.24)	0.088	0.11 (−0.14–0.36)	0.373	‐0.01 (−0.13–0.10)	0.836	0.19 (0.02–0.36)	0.033
	3rd quartile [47.45–52.54 μg/m^3^]	−0.02 (−0.14–0.10)	0.739	−0.28 (−0.53–0.04)	0.023	−0.07 (−0.18–0.04)	0.213	−0.01 (−0.18–0.16)	0.891
	4th quartile [52.56–64.01 μg/m^3^]	−0.03 (−0.16–0.10)	0.627	0.01 (−0.24–0.26)	0.920	−0.07 (−0.19–0.04)	0.217	0.10 (−0.08–0.27)	0.287
	Interquartile increase^1^	−0.02 (−0.06–0.02)	0.246	−0.04 (−0.12–0.04)	0.320	−0.03 (−0.07–0.01)	0.127	0.01 (−0.05–0.06)	0.822
PM_2.5_	1st quartile [20.65–24.03 μg/m^3^]	Reference		Reference		Reference		Reference	
	2nd quartile [24.03–25.61 μg/m^3^]	0.02 (−0.11–0.16)	0.723	0.01 (−0.26–0.28)	0.961	−0.01 (−0.13–0.11)	0.875	−0.18 (−0.36–0.01)	0.058
	3rd quartile [25.62–27.98 μg/m^3^]	−0.04 (−0.17–0.09)	0.543	−0.12 (−0.37–0.14)	0.378	0.06 (−0.05–0.18)	0.282	−0.10 (−0.28–0.07)	0.257
	4th quartile [28.02–35.82 μg/m^3^]	−0.02 (−0.15–0.11)	0.739	−0.23 (−0.49–0.03)	0.086	0.03 (−0.08–0.15)	0.554	−0.03 (−0.21–0.14)	0.720
	Interquartile increase^1^	−0.01 (−0.05–0.03)	0.529	−0.08 (−0.16–0.00)	0.057	0.02 (−0.02–0.05)	0.350	0.00 (−0.06–0.05)	0.940

Data represent the results of linear mixed model analysis for longitudinal cognitive scores using air pollutants, year, and the interaction term between air pollutants and year (air pollutants × year) as predictors. Covariates included age, sex, education, *APOE4* carrier status, and baseline CDR. Air pollutants were treated as categorical variables based on the quartile distribution, and the lowest quartile group was used as a reference.

*APOE4*, apolipoprotein E ε4; BMI, body mass index; CDR, Clinical Dementia Rating; CO, carbon monoxide; NO_2_, nitrogen dioxide; PM, particulate matter; SO_2_, sulfur dioxide.

^1^ Air pollutant quartiles were treated as ordinal variables.

^2^ Significant after multiple comparison corrections across five air pollutants using the false discovery rate method.

### Effects of chronic exposure to air pollution and *APOE4* on cognitive decline rates

To understand whether the effect of air pollution on cognitive decline rate was modified by *APOE4* carrier, we first conducted LMMs for longitudinal cognitive scores using three‐way interaction term between exposure, time, and *APOE4* (Table 4). Higher exposure to PM_2.5_ and APOE4 carrier status was associated with a faster rate of decline in visuospatial domain (*β* = −94.21, 95% CI = −155.98 to −32.44, *p* = 0.003).

**Table 4:** Effects of chronic exposure to air pollutants and *APOE4* carrier status on the rate of cognitive declines.

Predictor	Language domain		Visuospatial domain		Memory domain		Executive domain	
	*β* (95% CI)	*p*	*β* (95% CI)	*p*	*β* (95% CI)	*p*	*β* (95% CI)	*p*
CO × *APOE4* × year	0.49 (−0.29–1.26)	0.224	0.48 (−1.11–2.08)	0.558	−0.20 (−0.90–0.51)	0.587	0.62 (−0.47–1.71)	0.266
NO_2_ × *APOE4* × year	6.54 (−3.59–16.67)	0.208	4.07 (−16.72–24.87)	0.702	−5.24 (−14.49–4.01)	0.269	6.88 (−7.40–21.15)	0.348
SO_2_ × *APOE4* × year	17.57 (−88.32–123.69)	0.746	29.83 (−182.92–242.75)	0.785	−23.60 (−117.72–70.33)	0.622	43.62 (−99.97–187.16)	0.552
PM_10_ × *APOE4* × year	−7.27 (−23.84–9.28)	0.393	−16.11 (−49.85–17.47)	0.351	−5.14 (−20.11–9.85)	0.504	1.02 (−22.18–24.24)	0.932
PM_2.5_ × *APOE4* × year	−13.85 (−45.40–17.71)	0.394	−94.21 (−155.98–32.44)	0.003^1^	−14.59 (−42.21–13.05)	0.306	−30.59 (−73.44–12.28)	0.166

Data represent the results of linear mixed model analysis for longitudinal cognitive scores using a three‐way interaction term between air pollutants, *APOE4* carrier status, and year (air pollutants × *APOE4* × year) as a predictor. The covariates included age, sex, education, and baseline CDR.

*APOE4*, apolipoprotein E ε4; BMI, body mass index; CDR, Clinical Dementia Rating; CO, carbon monoxide; NO_2_, nitrogen dioxide; PM, particulate matter; SO_2_, sulfur dioxide.

^1^ Significant after multiple comparison corrections across five air pollutants using the false discovery rate method.

We further investigate the effect of air pollution on cognitive decline rate in *APOE4* carrier and noncarrier subgroups (Table S10). Higher exposure to PM2.5 was associated with a faster rate of decline in visuospatial domain only in *APOE4* carrier group (*β* = −94.21, 95% CI = −155.98 to −32.44, *p* = 0.003).

## Discussion

In this study, we assessed the effects of chronic exposure to air pollutants on the cognitive decline rate in AD patients with brain Aβ deposition and whose detailed demographic and socioeconomic information, residential history, comorbidities, and longitudinal neuropsychological evaluations were available. The major findings of this study are as follows: first, we found that higher 5‐year normalized hourly cumulative exposure to SO_2_ was associated with a faster rate of decline in the memory domain. Second, higher 5‐year normalized hourly cumulative exposure to PM_2.5_ was associated with a faster rate of decline in the visuospatial domain only in *APOE4* carriers. These associations remained statistically robust after adjusting for possible confounders, including age, sex, education, *APOE4* carrier status, baseline CDR, occupational history, smoking and alcohol history, BMI, and comorbidities, including hypertension, dyslipidemia, type 2 diabetes, and stroke. These findings suggest that chronic exposure to air pollutants could have deleterious effects on the clinical deterioration in the patients with AD.

Our first major finding was that higher 5‐year normalized hourly cumulative exposure to SO_2_ was associated with a faster rate of decline in the memory domain. This suggest that among the air pollutants, SO_2_ is important target for the management of AD. The detrimental effects of SO_2_ could be explained by neuroinflammation,^25^ synaptic dysfunction,^26^ and tau phosphorylation.^27^ Our finding is consistent with a previous study that showed the strongest effects of exposure to SO_2_ on CDR deterioration in patients with AD compared to exposure to CO, NO_2_, PM_10_, SO_2_, and ozone.^17^ Another study also showed detrimental effects of SO_2_ on the total and memory subdomain score of the MMSE in normal elderly population.^28^ However, most of previous studies evaluating the effect of air pollution on the risk of dementia or cognitive decline have been performed in normal elderly individuals without dementia from community‐based cohorts and most of these studies did not include SO_2_ as a predictor,^1^, ^4^, ^5^, ^6^, ^7^, ^11^, ^12^, ^29^, ^30^, ^31^, ^32^, ^33^ it may be possible that the effects of SO_2_ in AD were unrevealed in previous studies. Further studies are warranted to evaluate whether SO_2_ is associated with rapid clinical progression after AD occurrence rather than the occurrence of AD.

Our findings raise concerns about the current guidelines on the exposure limit of SO_2_ and suggest that dementia patients may need disease‐specific guidelines. As the highest SO_2_ exposure quartile (5.70–9.43 ppb/h) group showed faster decline of memory function than the lowest quartile group (2.40–4.80 ppb/h), current guidelines for general population may not be safe for dementia patients. The US Environmental Protection Agency National Ambient Air Quality Standards value for SO_2_ is 75 ppb (1‐h concentration),^34^ that according to a general guideline of the World Health Organization is 125 μg/m^3^ (24‐h mean concentration),^35^ and that as per the Korean Ministry of Environment Framework Act On Environmental Policy is 150 ppb (1‐h concentration),^36^ all of which are 7–15 times higher than the level of SO_2_ in the highest exposure group.

Our second major finding was that *APOE4* status and chronic exposure to PM_2.5_ were interactively associated with a faster decline in the visuospatial domain score. This result is consistent with previous longitudinal studies showing a significant interaction between PM_2.5_ and *APOE4* on cognitive deterioration.^32^, ^37^ Given that PM_2.5_ penetrates the BBB by disrupting tight junctions^38^ and that *APOE4* accelerates the breakdown of the BBB,^39^ aggravated disruption of BBB integrity could explain the interaction effect between PM_2.5_ and *APOE4* on the longitudinal decline in visuospatial domain score. However, the previous studies included community‐dwelling elderly individuals without dementia^32^, ^37^; moreover, the interaction effect was significant for all cognitive domains, including memory, language, and executive function,^32^ and all air pollutants, including not only PM_2.5_ ^32^, ^37^ but also NO_2_ and PM_10_.^32^ Considering that PM is also associated with an increased risk of Aβ accumulation in elderly individuals without dementia^16^ and animal models,^37^ the limited variance of Aβ due to the inclusion of cognitively impaired patients with confirmed Aβ deposition in our study could explain the discrepancy.

This study has several limitations. First, it included subjects from a tertiary referral center in Seoul, a metropolitan city in Korea, and 63.2% of the subjects had lived in Seoul for at least 5 years prior to baseline. Thus, various demographic features related to the residential environment may have been major confounders. Although the sensitivity analyses with further adjustment for living in Seoul showed similar results (data not shown), careful interpretation of our results in this context is required. Second, we included patients only if their residential addresses were located within 15 km of air monitoring stations. While such an inclusion criterion has been found appropriate for other diseases,^22^ dementia may need further examination to identify disease‐related optimal distance. Further evaluation using satellite‐based air pollutant measurement data would be required. Despite these limitations, our study shows a strong association between chronic exposure to air pollution and faster cognitive decline in patients with AD. Our findings emphasize that prompt consideration of air pollution is needed in the management of patients with AD from the policy‐making perspective as well as in clinical settings.

## Author Contributions

Young‐gun Lee, Seon‐Jin Yoon, and Byoung Seok Ye contributed to the conception and design of the study; Young‐gun Lee and Seon‐Jin Yoon contributed to the acquisition and analysis of data; Young‐gun Lee, Seon‐Jin Yoon, So Hoon Yoon, Sung Woo Kang, Seun Jeon, Minseok Kim, Dong Ah Shin, Chung Mo Nam, and Byoung Seok Ye contributed to drafting the text or preparing the figures.

## Conflict of Interest

The authors report no competing interests.

## Supporting information


Appendix S1.

## Data Availability

Data of air pollution level excluding address information and de‐identified clinical information supporting the findings of this study are available from the corresponding author upon reasonable request.
