# Supplementary material for: Air pollution is associated with faster cognitive decline in Alzheimer's disease
Source: Ann Clin Transl Neurol. 2023 Apr 27;10(6):964–73. doi: 10.1002/acn3.51779 (PMC10270255; doi:10.1002/acn3.51779)
Supplement: Supplementary file 1 — Appendix S1. [file ACN3-10-964-s001.docx]

**Supplementary Figure 1. Calculation of 5-year normalized hourly cumulative exposure values**

**
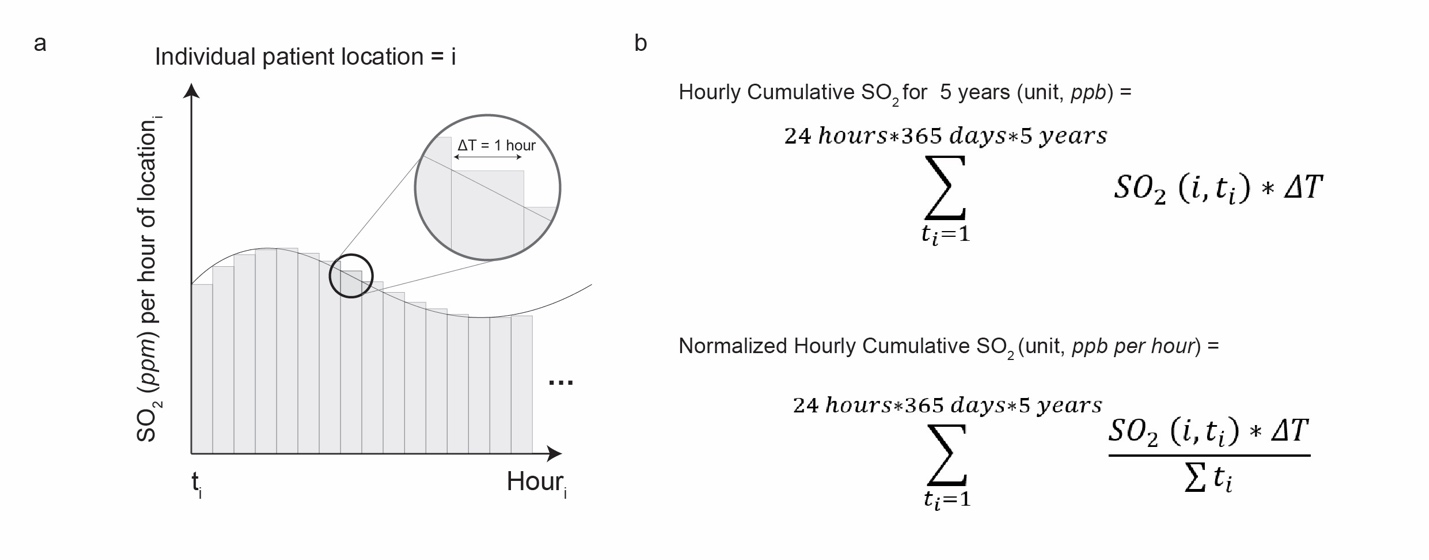
**

Abbreviations: SO_2_, sulfur dioxide. (a) Approximation of cumulative exposure to air pollutants in an individual who reside in location *i*. (b) Equation for 5-year hourly cumulative value and 5-year normalized hourly cumulative value.

**Supplementary Figure 2.** **Geographic distribution of the participants with chronic exposure to air pollutants**

**
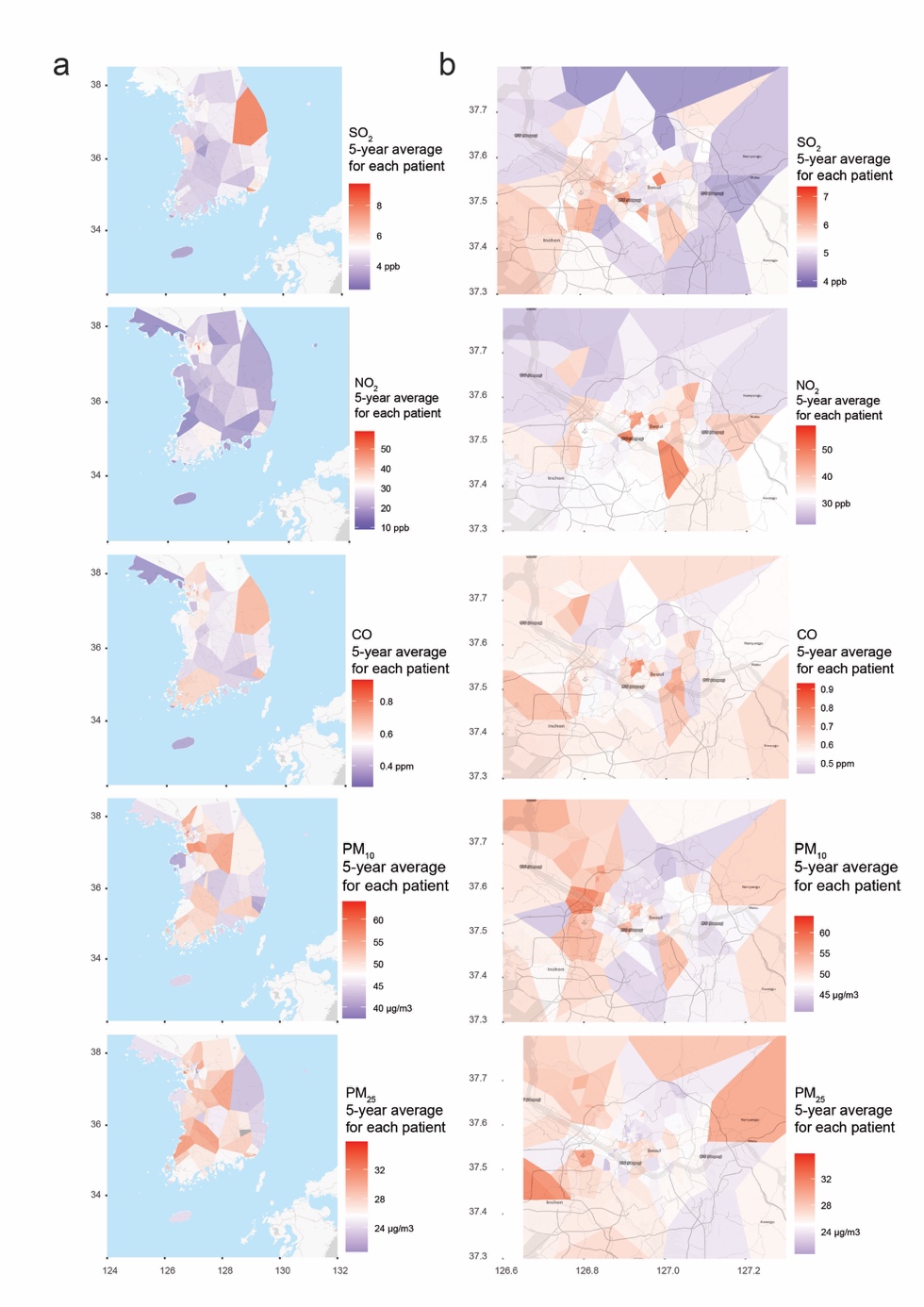
**

Abbreviations: CO, carbon monoxide; NO_2_, nitrogen dioxide; PM, particulate matter; SO_2_, sulfur dioxide. X-axis and Y-axis represent longitude and latitude, respectively. A. Five-year normalized hourly cumulative exposure to air pollutants in individual participants of this study (n = 269). B. Five-year normalized hourly cumulative exposure to air pollutants in individual participants who live in the near-capital area (n = 218). All the participant data were depicted using a Euclidean-Voronoi visualization algorithm.

**Supplementary Figure 3. Decline rate of cognitive domains in the participants with the lowest and highest SO_2_ exposure groups**


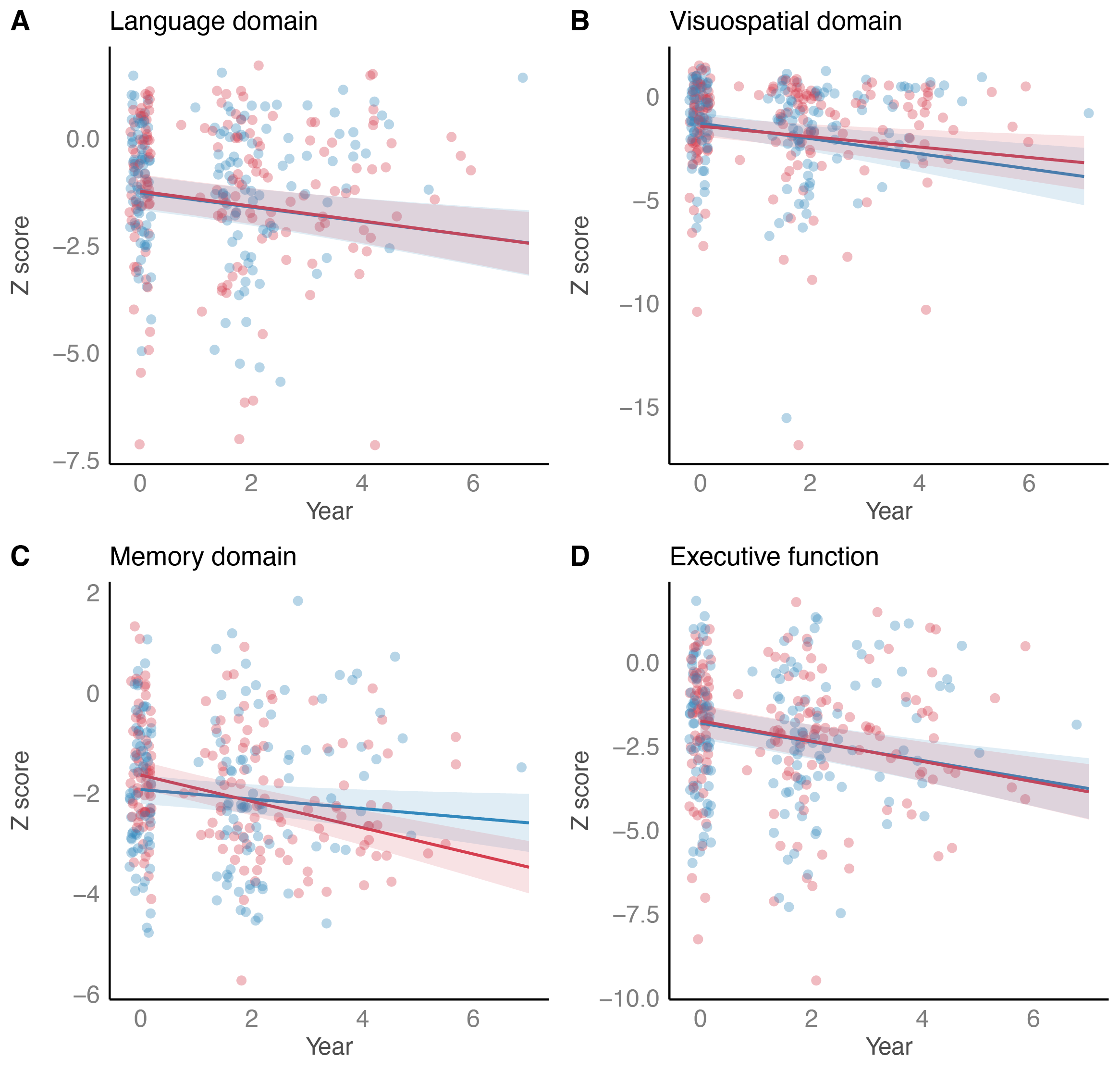


Abbreviations: *APOE4*, apolipoprotein E ε4; BMI, body mass index; CDR, Clinical Dementia Rating®; SO_2_, sulfur dioxide.

Data are the results of linear mixed model analysis for longitudinal cognitive scores using air pollutants, year, and the interaction term between air pollutants and year (air pollutants*year) as predictors. The covariates included age, sex, education, *APOE4* carrier status, and baseline CDR.

**Supplementary Table 1. Intercorrelation between 5-year normalized hourly cumulative exposure to air pollutants**

|  | **CO** | **NO_2_** | **SO_2_** | **PM_10_** |
| --- | --- | --- | --- | --- |
| **CO** |  |  |  |  |
| **NO_2_** | 0.74^†^ |  |  |  |
| **SO_2_** | 0.25^†^ | 0.45^†^ |  |  |
| **PM_10_** | 0.40^†^ | 0.31^†^ | 0.26^†^ |  |
| **PM_2.5_** | 0.11 | -0.16^††^ | -0.06 | 0.53 |

Abbreviations: CO, carbon monoxide; NO_2_, nitrogen dioxide; PM, particulate matter; SO_2_, sulfur dioxide.

Data represents Pearson's correlation coefficient (*r*).

^†^*p* < 0.001

^††^*p* < 0.05

**Supplementary Table 2. Effects of chronic exposure to air pollutant on the baseline cognitive scores**

|  |  | Model 1 | | Model 2 | | Model 3 | |
| --- | --- | --- | --- | --- | --- | --- | --- |
| Cognitive domain | Predictor | β (95% CI) | *P* | β (95% CI) | *P* | β (95% CI) | *P* |
| Language | CO | 0.49 (-0.91 – 1.89) | 0.492 | 0.67 (-0.79 – 2.13) | 0.365 | 0.65 (-0.81 – 2.11) | 0.382 |
|  | NO_2_ | 9.62 (-7.67 – 26.92) | 0.274 | 12.75 (-5.68 – 31.17) | 0.174 | 11.85 (-7.00 – 30.71) | 0.217 |
|  | SO_2_ | 75.74 (-115.68 – 267.16) | 0.437 | 81.74 (-121.35 – 284.82) | 0.429 | 67.76 (-139.04 – 274.56) | 0.519 |
|  | PM_10_ | 3.62 (-25.43 – 32.68) | 0.806 | 3.03 (-26.96 – 33.01) | 0.843 | 2.03 (-27.87 – 31.93) | 0.894 |
|  | PM_2.5_ | 10.51 (-45.53 – 66.55) | 0.712 | 10.62 (-46.84 – 68.08) | 0.716 | 16.79 (-41.37 – 74.96) | 0.570 |
| Visuospatial | CO | -0.03 (-1.90 – 1.83) | 0.972 | 0.19 (-1.76 – 2.15) | 0.847 | 0.26 (-1.72 – 2.24) | 0.796 |
|  | NO_2_ | 7.23 (-15.80 – 30.27) | 0.537 | 9.44 (-15.26 – 34.14) | 0.452 | 11.67 (-13.89 – 37.23) | 0.369 |
|  | SO_2_ | 57.97 (-196.74 – 312.69) | 0.654 | 25.24 (-246.61 – 297.08) | 0.855 | 38.94 (-241.12 – 319.00) | 0.784 |
|  | PM_10_ | -27.52 (-65.88 – 10.85) | 0.159 | -28.75 (-68.67 – 11.16) | 0.157 | -28.08 (-68.39 – 12.24) | 0.171 |
|  | PM_2.5_ | -54.88 (-128.29 – 18.53) | 0.142 | -57.36 (-133.42 – 18.70) | 0.139 | -56.32 (-134.07 – 21.43) | 0.155 |
| Memory | CO | 1.02 (-0.11 – 2.14) | 0.076 | 0.88 (-0.26 – 2.03) | 0.130 | 0.88 (-0.28 – 2.04) | 0.137 |
|  | NO_2_ | 5.32 (-8.64 – 19.28) | 0.454 | 2.69 (-11.88 – 17.26) | 0.717 | 1.69 (-13.34 – 16.72) | 0.825 |
|  | SO_2_ | 116.60 (-37.25 – 270.45) | 0.137 | 59.63 (-100.41 – 219.68) | 0.464 | 46.15 (-118.20 – 210.50) | 0.581 |
|  | PM_10_ | 10.39 (-12.92 – 33.71) | 0.381 | 6.71 (-16.90 – 30.31) | 0.576 | 6.49 (-17.26 – 30.23) | 0.591 |
|  | PM_2.5_ | -15.68 (-61.77 – 30.42) | 0.504 | -19.00 (-65.84 – 27.83) | 0.425 | -12.25 (-59.93 – 35.43) | 0.613 |
| Executive | CO | -0.03 (-1.85 – 1.79) | 0.974 | 0.40 (-1.46 – 2.26) | 0.673 | 0.39 (-1.49 – 2.27) | 0.681 |
|  | NO_2_ | 10.22 (-12.30 – 32.75) | 0.372 | 12.95 (-10.58 – 36.47) | 0.280 | 12.99 (-11.22 – 37.19) | 0.292 |
|  | SO_2_ | 30.35 (-218.97 – 279.68) | 0.811 | -40.71 (-299.89 – 218.48) | 0.757 | -58.66 (-323.97 – 206.64) | 0.664 |
|  | PM_10_ | -28.67 (-66.20 – 8.86) | 0.134 | -25.91 (-63.99 – 12.16) | 0.181 | -24.87 (-63.09 – 13.35) | 0.201 |
|  | PM_2.5_ | -26.97 (-100.91 – 46.97) | 0.473 | -22.09 (-96.74 – 52.56) | 0.560 | -19.05 (-94.64 – 56.54) | 0.620 |

Abbreviations: *APOE4*, apolipoprotein E ε4; BMI, body mass index; CDR, Clinical Dementia Rating®; CI, confidence interval; CO, carbon monoxide; NO_2_, nitrogen dioxide; PM, particulate matter; SO_2_, sulfur dioxide.

Data represents the results of general linear model for baseline cognitive scores using air pollutants as predictors. Model 1 was adjusted for age, sex, education, *APOE4* carrier status, and baseline CDR. Model 2: model 1 + further adjusted for the occupational history, insurance type, smoking and alcohol history, and BMI. Model 3: model 2 + further adjusted for comorbidities including hypertension, dyslipidemia, type 2 diabetes, and stroke.

**Supplementary Table 3. Effects of chronic exposure to SO_2_ on the rate of cognitive declines**

|  |  | Model 1 | | Model 2 | | Model 3 | |
| --- | --- | --- | --- | --- | --- | --- | --- |
| Cognitive domain | Predictor | β (95% CI) | *P* | β (95% CI) | *P* | β (95% CI) | *P* |
| Language | Year | -0.11 (-0.39 – 0.17) | 0.423 | -0.12 (-0.40 – 0.16) | 0.394 | -0.12 (-0.40 – 0.16) | 0.390 |
|  | SO_2_ | 73.58 (-116.59 – 263.69) | 0.453 | 74.61 (-120.83 – 269.77) | 0.472 | 65.38 (-131.94 – 262.52) | 0.536 |
|  | SO_2_*year | -10.18 (-62.92 – 42.42) | 0.705 | -8.56 (-61.13 – 43.97) | 0.750 | -8.33 (-60.84 – 44.22) | 0.756 |
| Visuospatial | Year | -0.41 (-0.99 – 0.15) | 0.153 | -0.44 (-1.02 – 0.12) | 0.128 | -0.44 (-1.02 – 0.12) | 0.128 |
|  | SO_2_ | 121.19 (-134.96 – 378.21) | 0.360 | 67.69 (-198.05 – 333.40) | 0.632 | 105.60 (-165.12 – 376.58) | 0.466 |
|  | SO_2_*year | 14.53 (-91.92 – 122.50) | 0.789 | 20.00 (-85.76 – 128.53) | 0.711 | 19.69 (-85.70 – 127.97) | 0.715 |
| Memory | Year | 0.15 (-0.10 – 0.40) | 0.232 | 0.15 (-0.10 – 0.40) | 0.231 | 0.15 (-0.09 – 0.41) | 0.226 |
|  | SO_2_ | 119.64 (-31.73 – 270.84) | 0.126 | 72.73 (-79.89 – 225.67) | 0.369 | 56.45 (-98.78 – 211.96) | 0.497 |
|  | SO_2_*year | -66.18 (-113.18 – -19.54) | 0.006 | -66.22 (-113.36 – -19.67) | 0.006 | -66.48 (-113.64 – -20.05) | 0.006 |
| Executive | Year | -0.18 (-0.56 – 0.20) | 0.356 | -0.19 (-0.57 – 0.19) | 0.332 | -0.19 (-0.57 – 0.19) | 0.331 |
|  | SO_2_ | 59.33 (-186.97 – 305.82) | 0.640 | 2.16 (-246.61 – 251.41) | 0.987 | -3.74 (-256.88 – 249.61) | 0.978 |
|  | SO_2_*year | -25.94 (-97.26 – 45.63) | 0.477 | -24.47 (-95.91 – 47.40) | 0.503 | -24.40 (-95.79 – 47.46) | 0.504 |

Abbreviations: *APOE4*, apolipoprotein E ε4; BMI, body mass index; CDR, Clinical Dementia Rating®; SO_2_, sulfur dioxide.

Data represent the results of linear mixed model analysis for longitudinal cognitive scores using SO_2_, year, and the interaction term between SO2 and year (SO_2_*year) as predictors. Model 1 was adjusted for age, sex, education, *APOE4* carrier status, and baseline CDR. Model 2: model 1 + further adjusted for the occupational history, insurance type, smoking and alcohol history, and BMI. Model 3: model 2 + further adjusted for comorbidities including hypertension, dyslipidemia, type 2 diabetes, and stroke.

**Supplementary Table 4. Effects of chronic exposure to CO on the rate of cognitive declines**

|  |  | Model 1 | | Model 2 | | Model 3 | |
| --- | --- | --- | --- | --- | --- | --- | --- |
| Cognitive domain | Predictor | β (95% CI) | *P* | β (95% CI) | *P* | β (95% CI) | *P* |
| Language | Year | -0.01 (-0.23 – 0.22) | 0.962 | 0.00 (-0.23 – 0.22) | 0.976 | 0.00 (-0.23 – 0.22) | 0.989 |
|  | CO | 0.54 (-0.86 – 1.93) | 0.456 | 0.80 (-0.60 – 2.21) | 0.283 | 0.79 (-0.61 – 2.18) | 0.294 |
|  | CO*year | -0.28 (-0.67 – 0.10) | 0.151 | -0.28 (-0.67 – 0.10) | 0.149 | -0.29 (-0.67 – 0.10) | 0.145 |
| Visuospatial | Year | -0.21 (-0.67 – 0.25) | 0.375 | -0.21 (-0.67 – 0.25) | 0.364 | -0.21 (-0.67 – 0.25) | 0.362 |
|  | CO | 0.32 (-1.58 – 2.21) | 0.745 | 0.64 (-1.27 – 2.57) | 0.528 | 0.76 (-1.16 – 2.69) | 0.462 |
|  | CO*year | -0.23 (-1.02 – 0.56) | 0.575 | -0.21 (-1.01 – 0.57) | 0.597 | -0.21 (-1.00 – 0.57) | 0.596 |
| Memory | Year | -0.03 (-0.24 – 0.17) | 0.750 | -0.03 (-0.24 – 0.17) | 0.753 | -0.03 (-0.24 – 0.17) | 0.750 |
|  | CO | 1.01 (-0.10 – 2.12) | 0.079 | 0.92 (-0.18 – 2.02) | 0.117 | 0.90 (-0.20 – 2.01) | 0.125 |
|  | CO*year | -0.28 (-0.64 – 0.07) | 0.116 | -0.29 (-0.64 – 0.07) | 0.114 | -0.28 (-0.64 – 0.07) | 0.116 |
| Executive | Year | -0.32 (-0.64 – -0.01) | 0.047 | -0.32 (-0.64 – -0.01) | 0.045 | -0.32 (-0.64 – -0.01) | 0.045 |
|  | CO | 0.16 (-1.66 – 1.97) | 0.866 | 0.64 (-1.15 – 2.44) | 0.500 | 0.65 (-1.15 – 2.45) | 0.498 |
|  | CO*year | 0.01 (-0.53 – 0.55) | 0.974 | 0.01 (-0.53 – 0.56) | 0.969 | 0.01 (-0.53 – 0.56) | 0.971 |

Abbreviations: *APOE4*, apolipoprotein E ε4; BMI, body mass index; CDR, Clinical Dementia Rating®; CO, carbon monoxide.

Data represent the results of linear mixed model analysis for longitudinal cognitive scores using CO, year, and the interaction term between CO and year (CO*year) as predictors. Model 1 was adjusted for age, sex, education, *APOE4* carrier status, and baseline CDR. Model 2: model 1 + further adjusted for the occupational history, insurance type, smoking and alcohol history, and BMI. Model 3: model 2 + further adjusted for comorbidities including hypertension, dyslipidemia, type 2 diabetes, and stroke.

**Supplementary Table 5. Effects of chronic exposure to NO_2_ on the rate of cognitive declines**

|  |  | Model 1 | | Model 2 | | Model 3 | |
| --- | --- | --- | --- | --- | --- | --- | --- |
| Cognitive domain | Predictor | β (95% CI) | *P* | β (95% CI) | *P* | β (95% CI) | *P* |
| Language | Year | -0.05 (-0.22 – 0.11) | 0.531 | -0.05 (-0.22 – 0.11) | 0.557 | -0.05 (-0.22 – 0.11) | 0.561 |
|  | NO_2_ | 8.28 (-9.00 – 25.54) | 0.353 | 12.47 (-5.34 – 30.24) | 0.188 | 11.79 (-6.29 – 29.83) | 0.223 |
|  | NO_2_*year | -3.58 (-8.53 – 1.37) | 0.157 | -3.65 (-8.57 – 1.30) | 0.148 | -3.65 (-8.57 – 1.30) | 0.147 |
| Visuospatial | Year | -0.51 (-0.85 – -0.17) | 0.003 | -0.50 (-0.84 – -0.17) | 0.004 | -0.50 (-0.85 – -0.17) | 0.004 |
|  | NO_2_ | 8.89 (-14.53 – 32.30) | 0.462 | 11.94 (-12.39 – 36.23) | 0.355 | 16.00 (-8.93 – 40.88) | 0.230 |
|  | NO_2_*year | 5.28 (-4.78 – 15.43) | 0.306 | 5.15 (-4.84 – 15.33) | 0.316 | 5.15 (-4.80 – 15.34) | 0.315 |
| Memory | Year | -0.06 (-0.22 – 0.09) | 0.406 | -0.07 (-0.22 – 0.09) | 0.396 | -0.07 (-0.22 – 0.09) | 0.395 |
|  | NO_2_ | 5.18 (-8.64 – 19.01) | 0.467 | 4.17 (-9.80 – 18.16) | 0.573 | 2.97 (-11.31 – 17.24) | 0.697 |
|  | NO_2_*year | -4.08 (-8.58 – 0.43) | 0.078 | -4.05 (-8.56 – 0.47) | 0.080 | -4.04 (-8.54 – 0.48) | 0.081 |
| Executive | Year | -0.36 (-0.59 – -0.13) | 0.003 | -0.36 (-0.60 – -0.13) | 0.002 | -0.36 (-0.60 – -0.13) | 0.002 |
|  | NO_2_ | 8.42 (-14.00 – 30.81) | 0.466 | 12.66 (-10.05 – 35.33) | 0.293 | 13.38 (-9.86 – 36.56) | 0.281 |
|  | NO_2_*year | 1.40 (-5.55 – 8.34) | 0.693 | 1.38 (-5.57 – 8.32) | 0.698 | 1.38 (-5.57 – 8.32) | 0.697 |

Abbreviations: *APOE4*, apolipoprotein E ε4; BMI, body mass index; CDR, Clinical Dementia Rating®; NO_2_, nitrogen dioxide.

Data represent the results of linear mixed model analysis for longitudinal cognitive scores using NO_2_, year, and the interaction term between NO2 and year (NO_2_*year) as predictors. Model 1 was adjusted for age, sex, education, *APOE4* carrier status, and baseline CDR. Model 2: model 1 + further adjusted for the occupational history, insurance type, smoking and alcohol history, and BMI. Model 3: model 2 + further adjusted for comorbidities including hypertension, dyslipidemia, type 2 diabetes, and stroke.

**Supplementary Table 6. Effects of chronic exposure to PM_10_ on the rate of cognitive declines**

|  |  | Model 1 | | Model 2 | | Model 3 | |
| --- | --- | --- | --- | --- | --- | --- | --- |
| Cognitive domain | Predictor | β (95% CI) | *P* | β (95% CI) | *P* | β (95% CI) | *P* |
| Language | Year | 0.10 (-0.30 – 0.51) | 0.623 | 0.10 (-0.30 – 0.51) | 0.615 | 0.10 (-0.30 – 0.51) | 0.616 |
|  | PM_10_ | 2.31 (-26.61 – 31.21) | 0.877 | 1.49 (-27.46 – 30.39) | 0.923 | 0.31 (-28.35 – 28.91) | 0.984 |
|  | PM_10_*year | -5.54 (-13.83 – 2.74) | 0.191 | -5.55 (-13.82 – 2.72) | 0.189 | -5.54 (-13.80 – 2.73) | 0.190 |
| Visuospatial | Year | 0.06 (-0.76 – 0.89) | 0.885 | 0.07 (-0.76 – 0.89) | 0.876 | 0.07 (-0.75 – 0.89) | 0.871 |
|  | PM_10_ | -22.51 (-61.59 – 16.57) | 0.264 | -22.82 (-62.21 – 16.56) | 0.276 | -22.17 (-61.52 – 17.18) | 0.293 |
|  | PM_10_*year | -8.18 (-25.07 – 8.62) | 0.342 | -8.18 (-25.10 – 8.49) | 0.339 | -8.25 (-25.17 – 8.39) | 0.335 |
| Memory | Year | 0.00 (-0.38 – 0.37) | 0.989 | 0.00 (-0.38 – 0.37) | 0.984 | 0.00 (-0.37 – 0.37) | 0.990 |
|  | PM_10_ | 9.38 (-13.71 – 32.50) | 0.431 | 5.77 (-16.93 – 28.50) | 0.632 | 5.48 (-17.17 – 28.16) | 0.651 |
|  | PM_10_*year | -3.96 (-11.50 – 3.62) | 0.305 | -3.94 (-11.49 – 3.64) | 0.307 | -3.97 (-11.52 – 3.61) | 0.304 |
| Executive | Year | -0.33 (-0.90 – 0.24) | 0.262 | -0.33 (-0.90 – 0.24) | 0.258 | -0.33 (-0.90 – 0.24) | 0.257 |
|  | PM_10_ | -26.44 (-63.77 – 10.93) | 0.170 | -24.02 (-60.81 – 12.77) | 0.219 | -23.38 (-60.12 – 13.34) | 0.234 |
|  | PM_10_*year | 0.19 (-11.39 – 11.81) | 0.975 | 0.21 (-11.36 – 11.85) | 0.971 | 0.22 (-11.35 – 11.85) | 0.971 |

Abbreviations: *APOE4*, apolipoprotein E ε4; BMI, body mass index; CDR, Clinical Dementia Rating®; PM, particulate matter.

Data represent the results of linear mixed model analysis for longitudinal cognitive scores using PM_10_, year, and the interaction term between PM_10_ and year (PM_10_*year) as predictors. Model 1 was adjusted for age, sex, education, *APOE4* carrier status, and baseline CDR. Model 2: model 1 + further adjusted for the occupational history, insurance type, smoking and alcohol history, and BMI. Model 3: model 2 + further adjusted for comorbidities including hypertension, dyslipidemia, type 2 diabetes, and stroke.

**Supplementary Table 7. Effects of chronic exposure to PM_2.5_ on the rate of cognitive declines**

|  |  | Model 1 | | Model 2 | | Model 3 | |
| --- | --- | --- | --- | --- | --- | --- | --- |
| Cognitive domain | Predictor | β (95% CI) | *P* | β (95% CI) | *P* | β (95% CI) | *P* |
| Language | Year | 0.16 (-0.26 – 0.58) | 0.441 | 0.17 (-0.26 – 0.58) | 0.433 | 0.17 (-0.25 – 0.59) | 0.428 |
|  | PM_2.5_ | 22.07 (-33.28 – 77.53) | 0.440 | 23.74 (-30.93 – 78.55) | 0.415 | 29.59 (-25.21 – 84.51) | 0.316 |
|  | PM_2.5_*year | -12.63 (-28.48 – 3.39) | 0.119 | -12.67 (-28.48 – 3.30) | 0.117 | -12.73 (-28.54 – 3.23) | 0.115 |
| Visuospatial | Year | 0.54 (-0.30 – 1.38) | 0.213 | 0.53 (-0.30 – 1.38) | 0.213 | 0.53 (-0.29 – 1.39) | 0.210 |
|  | PM_2.5_ | -39.86 (-112.84 – 33.19) | 0.291 | -39.78 (-112.46 – 33.37) | 0.306 | -41.60 (-115.21 – 32.54) | 0.296 |
|  | PM_2.5_*year | -32.42 (-64.61 – -0.67) | 0.048 | -32.06 (-64.40 – -0.79) | 0.048 | -32.24 (-64.72 – -1.06) | 0.047 |
| Memory | Year | -0.35 (-0.73 – 0.02) | 0.065 | -0.35 (-0.72 – 0.02) | 0.067 | -0.35 (-0.72 – 0.02) | 0.069 |
|  | PM_2.5_ | -13.52 (-58.57 – 31.62) | 0.561 | -16.34 (-60.71 – 28.13) | 0.489 | -7.64 (-52.40 – 37.27) | 0.751 |
|  | PM_2.5_*year | 6.65 (-7.41 – 20.84) | 0.356 | 6.52 (-7.53 – 20.72) | 0.365 | 6.46 (-7.59 – 20.66) | 0.369 |
| Executive | Year | -0.22 (-0.81 – 0.35) | 0.442 | -0.22 (-0.80 – 0.36) | 0.451 | -0.22 (-0.80 – 0.36) | 0.451 |
|  | PM_2.5_ | -11.00 (-83.74 – 61.99) | 0.770 | -7.09 (-77.98 – 64.03) | 0.851 | -3.82 (-75.35 – 67.99) | 0.921 |
|  | PM_2.5_*year | -2.92 (-24.71 – 19.15) | 0.790 | -3.15 (-25.05 – 18.93) | 0.773 | -3.16 (-25.07 – 18.91) | 0.773 |

Abbreviations: *APOE4*, apolipoprotein E ε4; BMI, body mass index; CDR, Clinical Dementia Rating®; PM, particulate matter.

Data represent the results of linear mixed model analysis for longitudinal cognitive scores using PM_2.5_, year, and the interaction term between PM_2.5_ and year (PM_2.5_*year) as predictors. Model 1 was adjusted for age, sex, education, *APOE4* carrier status, and baseline CDR. Model 2: model 1 + further adjusted for the occupational history, insurance type, smoking and alcohol history, and BMI. Model 3: model 2 + further adjusted for comorbidities including hypertension, dyslipidemia, type 2 diabetes, and stroke.

**Supplementary Table 8. Sensitivity analyses for the effects of chronic exposure to air pollutant on the rate of cognitive declines after adjustment of quantified amyloid deposition**

| Domain | Predictor | β (95% CI) | *p* |
| --- | --- | --- | --- |
| Language | CO*year | -0.28 (-0.67 – 0.10) | 0.152 |
|  | NO_2_*year | -3.58 (-8.52 – 1.36) | 0.156 |
|  | SO_2_*year | -12.68 (-65.40 – 39.89) | 0.637 |
|  | PM_10_*year | -5.78 (-14.06 – 2.49) | 0.172 |
|  | PM_2.5_*year | -12.68 (-28.49 – 3.31) | 0.117 |
| Visuospatial | CO*year | -0.24 (-1.03 – 0.55) | 0.553 |
|  | NO_2_*year | 5.04 (-5.03 – 15.20) | 0.329 |
|  | SO_2_*year | 10.24 (-96.39 – 118.26) | 0.850 |
|  | PM_10_*year | -8.45 (-25.34 – 8.36) | 0.326 |
|  | PM_2.5_*year | -32.69 (-64.83 – -0.97) | 0.046 |
| Memory | CO*year | -0.29 (-0.64 – 0.07) | 0.113 |
|  | NO_2_*year | -4.12 (-8.65 – 0.41) | 0.076 |
|  | SO_2_*year | -66.94 (-114.32 – -19.93) | 0.006^†^ |
|  | PM_10_*year | -4.02 (-11.61 – 3.59) | 0.300 |
|  | PM_2.5_*year | 6.59 (-7.56 – 20.86) | 0.363 |
| Executive | CO*year | 0.01 (-0.54 – 0.55) | 0.983 |
|  | NO_2_*year | 1.35 (-5.64 – 8.32) | 0.705 |
|  | SO_2_*year | -27.08 (-98.85 – 44.92) | 0.461 |
|  | PM_10_*year | 0.09 (-11.53 – 11.77) | 0.988 |
|  | PM_2.5_*year | -2.98 (-24.85 – 19.17) | 0.786 |

Abbreviations: BMI, body mass index; CDR, Clinical Dementia Rating®; CO, carbon monoxide; FBB, ^18^F-florbetaben; NO_2_, nitrogen dioxide; PET, positron emission tomography; PM, particulate matter; SO_2_, sulfur dioxide; SUVR, standardized uptake value ratio.

Data represent the results of linear mixed model analysis for longitudinal cognitive scores using air pollutants, year, and the interaction term between air pollutants and year (air pollutants*year) as predictors. Covariates included age, sex, education, baseline CDR, and global SUVR of FBB PET.

**^†^**Significant after multiple comparison corrections across five air pollutants using the false discovery rate method.

**Supplementary Table 9. Multi-pollutant models for the effects of chronic exposure to air pollutants on the rate of cognitive declines**

|  |  | Multi-pollutant models | | | |
| --- | --- | --- | --- | --- | --- |
|  |  | Four pollutants except NO_2_ | | Four pollutants except CO | |
| Domain | Predictor | β (95% CI) | *p* | β (95% CI) | *p* |
| Language | CO*year | -0.22 (-0.66 – 0.22) | 0.330 |  |  |
|  | NO_2_*year |  |  | -4.35 (-10.52 – 1.85) | 0.174 |
|  | SO_2_*year | -15.36 (-78.36 – 47.42) | 0.636 | 0.57 (-67.14 – 67.99) | 0.987 |
|  | PM_10_*year | -0.06 (-11.31 – 11.26) | 0.991 | 0.65 (-10.49 – 11.85) | 0.910 |
|  | PM_2.5_*year | -11.29 (-30.13 – 7.46) | 0.237 | -14.77 (-34.52 – 4.93) | 0.143 |
| Visuospatial | CO*year | -0.11 (-1.01 – 0.78) | 0.807 |  |  |
|  | NO_2_*year |  |  | 1.83 (-10.82 – 14.43) | 0.779 |
|  | SO_2_*year | 42.98 (-86.05 – 172.25) | 0.515 | 35.09 (-102.76 – 173.44) | 0.620 |
|  | PM_10_*year | 1.94 (-20.84 – 24.83) | 0.869 | -0.49 (-23.12 – 22.21) | 0.967 |
|  | PM_2.5_*year | -32.85 (-70.58 – 4.51) | 0.091 | -30.45 (-70.18 – 8.92) | 0.136 |
| Memory | CO*year | -0.15 (-0.53 – 0.23) | 0.459 |  |  |
|  | NO_2_*year |  |  | -0.97 (-6.38 – 4.43) | 0.729 |
|  | SO_2_*year | -73.73 (-127.94 – -19.66) | 0.009 | -70.83 (-129.00 – -12.80) | 0.020 |
|  | PM_10_*year | -3.74 (-13.42 – 5.94) | 0.455 | -4.55 (-14.19 – 5.09) | 0.362 |
|  | PM_2.5_*year | 10.43 (-5.33 – 26.18) | 0.202 | 10.01 (-6.65 – 26.66) | 0.246 |
| Executive | CO*year | 0.16 (-0.44 – 0.77) | 0.600 |  |  |
|  | NO_2_*year |  |  | 2.02 (-6.53 – 10.57) | 0.647 |
|  | SO_2_*year | -25.81 (-111.75 – 60.06) | 0.560 | -32.81 (-124.36 – 58.66) | 0.487 |
|  | PM_10_*year | 0.53 (-14.85 – 15.96) | 0.947 | 0.82 (-14.43 – 16.09) | 0.917 |
|  | PM_2.5_*year | -4.01 (-29.43 – 21.30) | 0.755 | -2.64 (-29.24 – 23.87) | 0.845 |

Abbreviations: *APOE4*, apolipoprotein E ε4; BMI, body mass index; CDR, Clinical Dementia Rating®; CO, carbon monoxide; NO_2_, nitrogen dioxide; PM, particulate matter; SO_2_, sulfur dioxide.

Data represent the results of linear mixed model analysis for longitudinal cognitive scores using air pollutants, year, and the interaction term between air pollutants and year (air pollutants*year) as predictors. Effects of multiple air pollutants were simultaneously considered. Covariates included age, sex, education, *APOE4* carrier status, and baseline CDR.

**Supplementary Table 10. Effects of chronic exposure to air pollutant on the rate of cognitive declines in the subgroup of *APOE4* carrier and non-carrier**

|  |  | *APOE4* carrier | | *APOE4* non-carrier | |
| --- | --- | --- | --- | --- | --- |
| Cognitive domain | Predictor | β (95% CI) | *P* | β (95% CI) | *P* |
| Language | CO*year | -0.10 (-0.60 – 0.40) | 0.682 | -0.57 (-1.13 – 0.00) | 0.050 |
|  | NO_2_*year | -0.43 (-6.58 – 5.73) | 0.891 | -7.77 (-15.51 – 0.02) | 0.052 |
|  | SO_2_*year | 6.51 (-65.64 – 78.21) | 0.860 | -13.71 (-86.86 – 59.75) | 0.714 |
|  | PM_10_*year | -8.47 (-20.10 – 3.09) | 0.155 | -2.25 (-13.59 – 9.12) | 0.698 |
|  | PM_25_*year | -23.02 (-42.88 – -3.06) | 0.028 | -4.08 (-26.82 – 18.88) | 0.726 |
| Visuospatial | CO*year | 0.00 (-1.11 – 1.11) | 0.997 | -0.41 (-1.56 – 0.74) | 0.488 |
|  | NO_2_*year | 7.20 (-6.05 – 20.50) | 0.291 | 2.56 (-13.28 – 18.45) | 0.752 |
|  | SO_2_*year | 27.96 (-131.08 – 188.69) | 0.732 | 5.79 (-139.35 – 155.32) | 0.937 |
|  | PM_10_*year | -18.10 (-43.59 – 7.18) | 0.163 | -0.78 (-23.41 – 21.80) | 0.946 |
|  | PM_25_*year | -81.36 (-127.90 – -36.15) | 0.001**^†^** | 16.99 (-25.43 – 58.98) | 0.431 |
| Memory | CO*year | -0.36 (-0.77 – 0.06) | 0.095 | -0.15 (-0.75 – 0.45) | 0.627 |
|  | NO_2_*year | -5.80 (-10.81 – -0.78) | 0.025 | -0.24 (-8.52 – 8.08) | 0.955 |
|  | SO_2_*year | -77.65 (-139.93 – -17.05) | 0.014 | -56.31 (-129.83 – 18.04) | 0.139 |
|  | PM_10_*year | -7.36 (-16.98 – 2.34) | 0.136 | -1.15 (-12.93 – 10.67) | 0.848 |
|  | PM_25_*year | -3.34 (-20.09 – 13.92) | 0.696 | 13.86 (-8.43 – 36.29) | 0.228 |
| Executive | CO*year | 0.28 (-0.42 – 0.97) | 0.438 | -0.40 (-1.26 – 0.48) | 0.362 |
|  | NO_2_*year | 3.84 (-4.61 – 12.24) | 0.372 | -2.92 (-14.90 – 9.02) | 0.633 |
|  | SO_2_*year | -4.85 (-106.61 – 96.12) | 0.925 | -49.48 (-150.80 – 55.02) | 0.344 |
|  | PM_10_*year | 0.83 (-15.24 – 16.92) | 0.920 | -0.28 (-17.21 – 16.90) | 0.974 |
|  | PM_25_*year | -18.18 (-46.95 – 10.99) | 0.216 | 13.14 (-20.54 – 47.71) | 0.434 |

Abbreviations: *APOE4*, apolipoprotein E ε4; BMI, body mass index; CDR, Clinical Dementia Rating®; CO, carbon monoxide; NO_2_, nitrogen dioxide; PM, particulate matter; SO_2_, sulfur dioxide.

Data represent the results of linear mixed model analysis for longitudinal cognitive scores using air pollutants, year, and the interaction term between air pollutants and year (air pollutants*year) as predictors in the subgroup of *APOE4* carriers. Covariates included age, sex, education, and baseline CDR.

**^†^**Significant after multiple comparison corrections across five air pollutants using the false discovery rate method.
